# The Associations Between Illness Perceptions and Expectations About Return to Work of Workers With Chronic Diseases and Their Significant Others: A Dyadic Analysis

**DOI:** 10.1007/s10926-022-10062-7

**Published:** 2022-08-17

**Authors:** N. C. Snippen, H. J. de Vries, C. A. M. Roelen, S. Brouwer, M. Hagedoorn

**Affiliations:** 1 Department of Health Sciences, Community and Occupational Medicine, University of Groningen, University Medical Center Groningen, PO Box 196, Groningen, 9700 AD The Netherlands; 2 Arbo Unie, Nieuwegein, The Netherlands; 3 Department of Health Sciences, Health Psychology, University of Groningen, University Medical Center Groningen, Groningen, The Netherlands

**Keywords:** Interpersonal Relations, Chronic Disease, Psychological Adaptation, Return to Work

## Abstract

*Purpose* To examine the associations between illness perceptions and expectations about full return to work (RTW) of workers with chronic diseases and their significant others. *Methods* This study used cross-sectional data of 94 dyads consisting of workers with chronic diseases and their significant others. We performed dyadic analyses based on the Actor-Partner Interdependence Model (APIM), estimating associations of illness perceptions of the two members of the dyad with their own expectations about the worker’s full RTW within six months (actor effect) as well as with the other dyad member’s expectations about the worker’s RTW (partner effect). *Results* Illness perceptions of one dyad member were significantly associated with his or her own RTW expectations (actor effect composite illness perceptions score; *B* = −0.05, *p* < .001; *r*_*d*_ = .37) and with the other dyad member’s RTW expectations (partner effect composite illness perceptions score; *B* = −0.04, *p* < .001; *r*_*d*_ = .35). That is, more negative illness perceptions of one member of the dyad were associated with more negative RTW expectations in both dyad members. For most illness perception domains, we found small to moderate actor and partner effects on RTW expectations (*r*_*d*_ range: .23–.44). *Conclusions* This study suggests that illness perceptions and RTW expectations should be considered at a dyadic level as workers and their significant others influence each other’s beliefs. When trying to facilitate adaptive illness perceptions and RTW expectations, involving significant others may be more effective than an individualistic approach targeted at the worker only.

**Supplementary Information:**

The online version contains supplementary material available at 10.1007/s10926-022-10062-7.

## Introduction

Workers with chronic diseases are at higher risk of involuntary early labor market exit because of work disability and unemployment as compared to workers without a chronic disease [1, 2]. In recent years, increasing attention has been paid to the role of illness perceptions in the context of work participation of workers with chronic diseases [3–7]. Previous research has shown that negative perceptions of workers concerning the duration, consequences, emotional impact, treatment efficacy, personal control and understanding of the illness are associated with increased risks of involuntary early labor market exit across various chronic health conditions [3, 5–7]. In addition, illness perceptions of workers have been shown to be strongly related to expectations about return to work (RTW) [4], which is one of the strongest prognostic factors of work-related outcomes like RTW, duration of sick leave and disability benefit receipt [7–14].

There is increasing evidence that significant others like partners, family members or friends affect an individual’s illness perceptions, adaptation to chronic illness and work participation through their interactions with the person with the disease [15–18]. Rather than illness perceptions being developed in isolation, the perceptions of individuals with chronic diseases and their significant others are connected [15]. It has therefore been proposed that coping and adaptation to chronic disease should be viewed from a dyadic perspective, in which the significant other’s appraisals, responses and interactions with the person with the chronic disease are also taken into account [17–21]. There is already some evidence that illness perceptions and RTW expectations of both workers and their significant others might play an important role in work participation outcomes of workers with persistent back pain [22, 23]. For instance, one study suggests that pessimistic beliefs about the likelihood of RTW of disability benefit claimants and their significant others may act as obstacles to work participation [22]. Another study found that couples in which the worker had become incapacitated for work had more negative perceptions about the consequences of the worker’s persistent back pain than couples in which the worker had remained in work despite persistent back pain [23]. However, the current level of evidence is low as the existing evidence is based on qualitative studies with relatively small study samples and quantitative knowledge on this topic is lacking. Moreover, to date the associations between illness perceptions and RTW expectations among workers with chronic diseases and their significant others has not been examined dyadically.

Gaining insight into effects of illness perceptions of workers and significant others on their RTW expectations could provide evidence-based recommendations regarding intrapersonal and interpersonal factors that can be targeted to modify RTW expectations in order to facilitate RTW [7–14]. Therefore, the aim of this study was to examine the associations between illness perceptions and RTW expectations of workers with chronic diseases and their significant others at a dyadic level. More specifically, we examined the associations of both the worker’s and his/her significant other’s illness perceptions with (i) a person’s own RTW expectations, and (ii) the other dyad member’s RTW expectations.

## Methods

### Study Design

This study used cross-sectional data of dyads consisting of workers and their significant others, which was collected for the purpose of this study and subjected to dyadic analysis [24].

### Participants and Inclusion Criteria

We included dyads consisting of workers who had been on sick leave due to a chronic health condition for at least two weeks, and one of their significant others (i.e., partner, family member or friend). To be eligible for participation, workers had to be between 18 and 65 years of age, be or recently have been on sick leave due to chronic health problems, and have a significant other who was willing to participate in the study (self-chosen by the worker). In addition, participants had to be proficient in written Dutch. Furthermore, the source population consisted of employees only, with self-employed workers falling beyond this population. The inclusion period lasted from June 2019 until September 2020.

### Procedure

We recruited participants through Arbo Unie, a large Dutch occupational health service (OHS). In the Netherlands, the OHS advises sick-listed workers and their employers about RTW. For this purpose, sick-listed workers are invited for a consultation with an occupational health physician within six weeks after the first registered day of sick leave. During the 15-month inclusion period, an extra paragraph was added to the invitation for this consultation, informing workers and their significant others about this study. In the added paragraph, a link was included to a dedicated webpage with more detailed study information and the online questionnaires for both the worker and significant other.

At the start of the questionnaire, participants were screened for eligibility and asked to give informed consent. Participants who did not meet the inclusion criteria or did not give informed consent were excluded and automatically directed to the end of the questionnaire. To minimize attrition due to missing values, automatic response requests were used to alert participants about any unanswered questions when moving to another page of the questionnaire.

The Central Ethics Review Board of the University Medical Center Groningen approved the study protocol (CTc UMCG 201,700,925). Participants received written information regarding the confidentiality and anonymity of the study results and were given an opportunity to ask questions. Informed consent was obtained from all participants.

### Measures

Workers and significant others individually completed a questionnaire that measured expectations about the worker’s RTW, illness perceptions and sociodemographic characteristics.

#### Primary Outcome

The primary outcome measure was expectations about the worker’s full RTW within six months, based on the ‘self-predicted certainty question’ of Heymans et al. [8]: “How certain are you that you will be fully back at work in six months?”. Workers answered the question on a 5-point scale: (1)“completely uncertain”, (2)“a little uncertain”, (3)“somewhat certain”, (4) “certain”, (5) “completely certain”. Full RTW was defined as working the contracted working hours [8]. Significant others answered the question “How certain are you that the worker will be fully back at work in six months?” on the same 5-point scale.

#### Illness Perceptions

We measured illness perceptions of workers and significant others with respectively the Dutch version of the Brief Illness Perception Questionnaire (IPQ-B) [25, 26] and a significant other version of the IPQ-B, which was adapted from the spouse version of the IPQ-R [27]. In this study, we used the first eight items of the IPQ-B which were measured on a 11-point scale (ranging from zero to ten). The eight items assessed the worker’s and significant other’s illness perceptions about: (1) the influence of the illness on the worker’s daily life (consequences), (2) the duration of the illness (illness duration), (3) the worker’s control over the illness (personal control), (4) the extent to which treatment can help with controlling the illness (treatment control), (5) the severity of the symptoms experienced by the worker (illness identity), (6) the worker’s concern about the illness (concern), (7) the worker’s emotional response to the illness (emotional response), and (8) the worker’s degree of understanding of the illness (illness coherence).

Higher scores on consequences, illness duration, illness identity, concern, and emotional response reflected more negative perceptions, while higher scores on personal control, treatment control, and illness coherence reflected more positive perceptions. A composite illness perceptions score was computed by summing up the scores of the eight items, with a reverse scoring of the items on personal control, treatment control and illness coherence. For this composite score, we person-mean imputed data for participants with missing data on no more than three items. A higher composite score reflected more negative perceptions. The Cronbach's alpha of the IPQ-B composite score in this study was 0.71 for workers and 0.74 for significant others, which is similar to what was found in previous research [28–31].

#### Covariates

Sociodemographic measures and data about workers’ and significant others’ perceived relationship quality was collected to describe the sample and potentially include as covariates. With regard to sociodemographic measures, we collected data about the workers’ age, gender, educational level (low, medium, or high), type of chronic disease (somatic, mental, mixed), and employment status (fulltime vs. parttime). In addition, data was collected about the significant others’ age, gender, educational level, chronic disease (yes/no), and their relationship with the worker (i.e., partner, parent, adult child, sibling, friend). Finally, we collected data from both workers and significant others about their perceived relationship quality with the other dyad member, using a relationship quality rating scale from 0 through 10, with zero representing the worst possible and ten the best possible relationship [32].

### Preliminary Analyses

Preliminary analyses were performed to ensure that there was no violation of the assumptions of normality and homogeneity of variance. In addition, we conducted a series of preliminary analyses to examine associations between demographic characteristics and the outcome variables (i.e., RTW expectations) to assess the need to include covariates in the analyses. Significant others’ age was significantly associated with their own expectations of the worker’s RTW (*r* = −0.329, *p* = 0.001). Gender, educational level, type of chronic disease, employment status, type of relationship with the other dyad member, and perceived relationship quality were not associated with dyad members’ RTW expectations.

### Dyadic Analyses

In preparation for the analyses, data was formatted in a pairwise structure in SPSS version 26 using the individual-to-pairwise macro from Kenny [33] and the predictor variables were grand-mean centered in accordance with the recommendations from Kenny et al. [24].

**Fig. 1:**
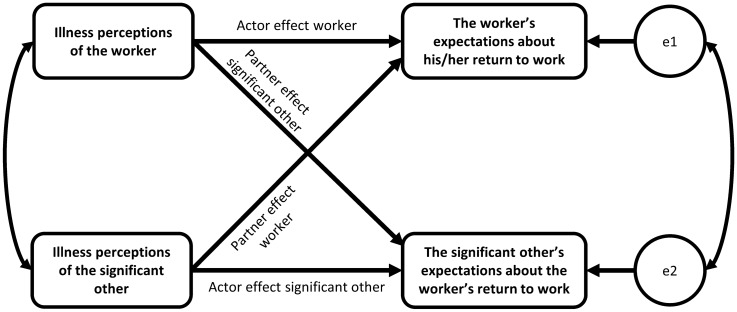
Actor-Partner Interdependence Model applied to this study. Conceptual Actor-Partner Interdependence Model depicting the examined actor and partner effects of illness perceptions on expectations about the worker's return to work within worker-significant other dyads

We performed dyadic analyses using the Actor-Partner Interdependence Model (APIM) [24] to determine dyadic associations between illness perceptions of workers and significant others (i.e., independent variable) and their expectations about the worker’s full RTW within six months (i.e., outcome variable). Interdependence means that the responses from the two individuals within a dyad are linked (i.e., non-independent). APIM analysis allows researchers to model the non-independence in the two dyad members’ responses by measuring the associations between their scores, as well as their intrapersonal (i.e., actor) effects and interpersonal (i.e., partner) effects [34]. Thus, in this study, worker and significant other expectations about the worker’s full RTW within six months were regressed on their own illness perceptions (i.e., actor effect) as well as on their counterpart’s illness perceptions (i.e., partner effect). Figure 1 displays the APIM framework applied to this study. We ran separate analyses for the composite illness perceptions score and each of the eight illness perception domains.

The analyses consisted of four steps, in which the two-intercept method of Multilevel Modeling was applied [24, 35]. In the first step the full APIM was estimated. To increase statistical power and simplify the models, we tested for differences in coefficients between dyad members (step 2) and, when appropriate, tested more parsimonious models in which intercepts, actor effects and partner effects that did not differ between workers and significant others were constrained to be equal for dyad members (step 3). Finally, in the fourth step, correlation coefficient effect sizes (*r*_*d*_) were estimated for the statistically significant effects in the final models. Each of the four analyses steps is described in more detail below. Furthermore, an example syntax for the first three steps is provided in Online Resource 1.

#### Step 1: Estimating the Full APIM

In the first step, the full APIM was estimated including an intercept, actor effect and partner effect for each dyad member (i.e., the estimated model included two intercepts, two actor effects and two partner effects). A total of nine APIMs were conducted to test whether illness perceptions of workers and significant others were significantly associated with a dyad member’s own expectations about the worker’s RTW (actor effect) and the other member’s RTW expectations (partner effect). To account for the interdependence between dyad members’ scores, the actor and partner effects were estimated simultaneously and the correlations of dyad members’ predictor and outcome variables, respectively, were also modeled. The models controlled for workers’ and significant others’ age.

#### Step 2: Testing for Differences Between Dyad Members

In the second step, contrast analyses were used to examine whether there were statistically meaningful differences between dyad members in the estimated intercepts, actor effects and partner effects. More specifically, we tested whether the intercepts, actor effects and partner effects significantly differed between workers and significant others (i.e., to examine whether actor effects and partner effects were stronger for one of the dyad members) or whether they could be considered to be equal for both dyad members. The findings of this step were used to develop more parsimonious models in step 3.

#### Step 3: Estimating Average Intercepts and Effects Across Dyad Members

Based on the results obtained in the second step, in the third step, we tested more parsimonious models in which, when appropriate, the intercepts, actor effects and partner effects were constrained to be equal for dyad members. In addition to developing more parsimonious models with fewer beta coefficients, an important advantage of constraining the coefficients to be equal for dyad members is an increase in statistical power as the scores of both dyad members are used to estimate average beta coefficients (i.e., the number of observations used for each beta coefficient is doubled). We therefore estimated average beta coefficients across dyad members for the intercepts, actor effects and partner effects that could be considered to be equal for workers and significant others, and tested whether the average actor effects and partner effects were significantly associated with RTW expectations. The intercepts, actor-effects and partner effects that were statistically different between workers and significant others (step two) remained as separate beta coefficients in the models. The final models could therefore include separate coefficients for workers and significant others, average coefficients or a combination of separate and average coefficients.

#### Step 4: Estimating Correlation Coefficient Effect Sizes

Finally, in the fourth step, we estimated correlation coefficient effect sizes (*r*_*d*_) for the statistically significant actor and partner effects in the final models [24]. Following the recommendations of Kenny et al. [24], we adjusted the effect sizes for the independent variables of which the scores of workers and significant others were strongly correlated (i.e., > 0.5 or < −0.5) to take into account the non-independence within dyads, and otherwise used the unadjusted effect sizes. We refer to the book of Kenny et al. [24] for more detailed information about determining the effect sizes in APIM analyses. Following the guidelines of Cohen [36], effects sizes of *r*_*d*_ = 0.1, *r*_*d*_ = 0.3, and *r*_*d*_ = 0.5 were considered to be small, medium and large in magnitudes, respectively.

## Results

A total of 166 workers completed the questionnaire. Workers for whom there was no data available from a significant other were excluded from the analyses (n = 72). The final study sample consisted of 94 dyads of workers (56.6%) and their significant others. There were no statistically significant differences between included and excluded workers with regard to age, gender, educational level, type of disease, comorbid conditions, perceived relationship quality, illness perceptions and RTW expectations. The mean age of included workers was 53.7 years (*SD* = 9.9, range: 25–65 years). A small majority of workers was male (55.3%) and had a low or medium level of education (53.2%). Most workers (80.9%) indicated to have a somatic disease, particularly musculoskeletal disorders (47.9%), cardiovascular disease (19.1%), neurological conditions (17.0%), and respiratory disease (14.9%). Furthermore, 36.2% of the workers had a mental illness, and almost half of the workers (44.7%) had comorbid conditions. The mean age of significant others was 52.6 years (*SD* = 13.4, range: 20–96 years), the majority was the partner or spouse of the worker (88.3%) (Table 1).

**Table 1:** Participant characteristics (*N* = 94 dyads)

Characteristic	Workers		Significant others	
Age in years (*SD*)	53.7	(9.9)	52.6	(13.4)
Gender				
Male	52	(55.3%)	39	(41.5%)
Female	42	(44.7%)	55	(58.5%)
Educational level				
Low	17	(18.1%)	19	(20.2%)
Medium	33	(35.1%)	44	(46.8%)
High	43	(45.7%)	30	(31.9%)
Missing	1	(1.1%)	1	(1.1%)
Relation to worker				
Partner/spouse	–		83	(88.3%)
Parent	–		5	(5.3%)
Adult child	–		4	(4.3%)
Sibling	–		1	(1.1%)
Friend	–		1	(1.1%)
Relationship quality, mean (range)	8.7	(6–10)	8.6	(5–10)
Type of chronic disease				
Somatic	59	(62.8%)	37	(39.4%)
Mental	17	(18.1%)	5	(5.3%)
Mixed	17	(18.1%)	6	(6.4%)
None	–		45	(47.9%)
Missing	1	(1.1%)	1	(1.1%)
Number of chronic diseases				
0	–		45	(47.9%)
1	51	(54.3%)	27	(28.7%)
> 1	42	(44.7%)	21	(22.3%)
Missing	1	(1.1%)	1	(1.1%)
Employment status				
Fulltime (≥ 36 h per week)	59	(62.8%)	26	(27.7%)
Part-time (12—35 h per week)	35	(37.2%)	38	(40.4%)
Not employed (< 12 h per week)	–		29	(30.9%)
Missing	–		1	(1.1%)
Mean scores (*SD*)				
RTW expectations (scale 1–6)	3.0	(1.3)	3.0	(1.4)
Composite illness perceptions score (scale 0–80)	48.7	(10.2)	46.4	(10.5)
Consequences (scale 1–6)	7.7	(2.0)	7.4	(2.0)
Timeline (scale 0–10)	6.2	(3.0)	6.0	(3.0)
Personal control (scale 0–10)	4.1	(2.4)	4.8	(2.7)
Treatment control (scale 0–10)	6.8	(2.1)	7.3	(2.4)
Illness identity (scale 0–10)	7.6	(1.8)	7.2	(1.9)
Concern (scale 0–10)	6.5	(2.5)	7.0	(2.2)
Illness coherence (scale 0–10)	7.3	(2.4)	8.0	(2.0)
Emotional response (scale 0–10)	6.7	(2.4)	6.6	(2.5)

*SD* standard deviation

### Representativeness of the Study Sample

There was no data available on the number and characteristics of sick-listed workers who received the invitation but decided not to participate in this study. However, we were able to compare our sample with a large and representative cohort from Arbo Unie consisting of 3,729 workers with a chronic disease who were sick-listed between January 2020 and September 2021. The mean age of workers was considerably higher in our study (53.7 years, *SD* = 9.9) than in the larger cohort (40.4 years, *SD* = 15.9). Furthermore, compared to workers in that cohort, a higher percentage of workers in our study sample was male (55.3% vs. 33.6%), had a musculoskeletal disorder (47.9% vs. 34.5%) or a mental illness (36.2% vs. 24.4%).

### Correlations

The correlation coefficients of all variables are depicted in Table 2. We found strong correlations between workers’ and significant others’ composite illness perceptions scores (*r* = 0.64) and their expectations about the worker’s RTW (*r* = 0.77). While most of the correlations between their scores on the illness perception domains were moderate to strong (*r* ≥ 0.41), there were weak correlations between workers and significant others for the domains *illness identity* (*r* = 0.28) and *illness coherence* (*r* = 0.21). Workers’ and significant others’ composite illness perceptions scores and scores on the domains *consequences*, *timeline*, *treatment control*, and *concern* were significantly associated with both their own and the other dyad member’s certainty that the worker would be fully back at work in six months (*r* ≤ −0.27 or *r* ≥ 0.34).

**Table 2:** Intercorrelations of worker and significant other illness perceptions and RTW expectations (condensed table)

	Composite illness perceptions score	Consequences	Timeline	Personal control	Treatment control	Illness identity	Concern	Illness coherence	Emotional response	RTW expectations of significant others^b^	RTW expectations of workers^b^
Composite illness perceptions score	0.64^**^	0.78^**^	0.55^**^	−0.54^**^	−0.39^**^	0.59^**^	0.72^**^	−0.28^**^	0.69^**^	−0.48^**^	−0.50^**^
Consequences	0.67^**^	0.48^**^	0.41^**^	−0.33^**^	−0.18	0.53^**^	0.59^**^	−0.09	0.47^**^	−0.43^**^	−0.30^**^
Timeline	0.63^**^	0.27^*^	0.73^**^	−0.11	−0.22^*^	0.21^*^	0.26^*^	0.03	0.07	−0.44^**^	−0.46^**^
Personal control	−0.42^**^	−0.31^**^	−0.13	0.41^**^	0.44^**^	−0.33^**^	-0.18	0.11	−0.28^**^	0.41^**^	0.38^**^
Treatment control	−0.34^**^	−0.05	−0.22^*^	0.25^*^	0.62^**^	−0.01	0.00	0.09	−0.08	0.37^**^	0.37^**^
Illness identity	0.58^**^	0.67^**^	0.26^*^	−0.19	0.04	0.28^**^	0.53^**^	0.11	0.34^**^	−0.21^*^	−0.15
Concern	0.76^**^	0.55^**^	0.46^**^	−0.18	0.00	0.43^**^	0.47^**^	−0.02	0.67^**^	−0.27^**^	−0.31^**^
Illness coherence	−0.34^**^	0.04	−0.04	0.17	0.27^**^	0.15	−0.149	0.24^*^	−0.25^*^	−0.10	−0.09
Emotional response	0.66^**^	0.40^**^	0.21^*^	−0.06	0.01	0.42^**^	0.51^**^	−0.14	0.58^**^	−0.14	−0.21^*^
RTW expectations of workers^a^	−0.60^**^	−0.44^**^	−0.51^**^	0.19	0.39^**^	−0.32^**^	−0.44^**^	0.05	−0.15	0.77^**^	1
RTW expectations of significant others^a^	−0.57^**^	−0.46^**^	−0.45^**^	0.14	0.34^**^	−0.40^**^	−0.48^**^	0.09	−0.27^**^	1	0.77^**^

Correlations among workers are below the diagonal; correlations among significant others are above the diagonal; the diagonal depicts the correlations between workers and significant others. *p < 0.05; **p < 0.01. ^a^Correlations with illness perceptions of workers; ^b^Correlations with illness perceptions of significant others; RTW = return to work

### Actor and Partner Effects

An overview of the two-intercept models and the final models including effect sizes (*r*_*d*_) for all statistically significant actor and partner effects is provided in Table 3.

**Table 3:** Associations between illness perceptions and RTW expectations among dyads of workers and their significant others

	Two-intercept model^a^			*Sig*	Final model^b^					*r*_*d*_
	*B*	*SD*	*t*			*B*	*SD*	*t*	*Sig*	
Composite illness perceptions score (*N* = 94)										
Intercept worker	3.14	0.12	27.16	< 0.001**	Intercept	3.10	0.10	29.81	< 0.001**	
Intercept significant other	3.08	0.12	25.83	< 0.001**	Actor effect	−0.05	0.01	−5.80	< 0.001**	0.37^c^
Actor effect worker	−0.06	0.01	−4.43	< 0.001**	Partner effect	−0.04	0.01	−5.58	< 0.001**	0.35^c^
Actor effect significant other	−0.03	0.01	−1.74	0.084						
Partner effect worker	−0.03	0.01	−2.03	0.046*						
Partner effect significant other	−0.06	0.01	−4.02	< 0.001**						
Consequences (*N* = 94)										
Intercept worker	3.13	0.13	24.94	< 0.001**	Intercept	3.08	0.11	26.82	< 0.001**	
Intercept significant other	3.05	0.12	24.66	< 0.001**	Actor effect	−0.25	0.04	−5.92	< 0.001**	0.43
Actor effect worker	−0.29	0.07	−3.96	< 0.001**	Partner effect	−0.16	0.04	−3.79	< 0.001**	0.29
Actor effect significant other	−0.18	0.07	−2.63	0.010*						
Partner effect worker	−0.11	0.07	−1.54	0.127						
Partner effect significant other	−0.23	0.07	−3.18	0.002*						
Illness duration (*N* = 92)										
Intercept worker	3.08	0.12	25.24	< 0.001**	Intercept	3.06	0.11	26.78	< 0.001**	
Intercept significant other	3.05	0.13	24.08	< 0.001**	Actor effect	−0.13	0.03	−4.26	< 0.001**	0.29^c^
Actor effect worker	−0.16	0.06	−2.66	0.010*	Partner effect	−0.11	0.03	−3.74	< 0.001*	0.26^c^
Actor effect significant other	−0.11	0.06	−1.85	0.067						
Partner effect worker	−0.08	0.06	−1.44	0.155						
Partner effect significant other	−0.12	0.06	−1.85	0.067						
Personal control of the worker (*N* = 94)										
Intercept worker	3.02	0.13	22.42	< 0.001**	Intercept	2.98	0.12	24.13	< 0.001**	
Intercept significant other	2.95	0.13	21.99	< 0.001**	Actor effect worker	0.03	0.06	0.42	0.673	ns
Actor effect worker	0.02	0.06	0.30	0.767	Actor effect significant other	0.22	0.05	4.41	< 0.001**	0.44
Actor effect significant other	0.22	0.05	4.12	< 0.001**	Partner effect worker	0.19	0.05	3.61	0.001**	0.37
Partner effect worker	0.19	0.05	3.61	0.001*	Partner effect significant other	−0.02	0.06	−0.33	0.746	ns
Partner effect significant other	−0.02	0.06	−0.35	0.728						
Treatment control (*N* = 94)										
Intercept worker	3.09	0.13	23.47	< 0.001^**^	Intercept	3.06	0.12	24.97	< 0.001^**^	
Intercept significant other	3.04	0.13	22.56	< 0.001^**^	Actor effect	0.15	0.04	3.82	< 0.001^**^	0.26^c^
Actor effect worker	0.16	0.08	2.13	0.036^*^	Partner effect	0.13	0.04	3.38	0.001^**^	0.23^c^
Actor effect significant other	0.15	0.07	2.08	0.040^*^						
Partner effect worker	0.12	0.07	1.76	0.082						
Partner effect significant other	0.11	0.08	1.43	0.157						
Illness identity (*N* = 94)										
Intercept worker	3.13	0.14	23.05	< 0.001^**^	Intercept	3.10	0.12	24.85	< 0.001^**^	
Intercept significant other	3.08	0.13	23.44	< 0.001^**^	Actor effect	−0.17	0.05	−3.60	0.005^**^	0.30
Actor effect worker	−0.25	0.08	−3.18	0.002^*^	Partner effect worker	−0.04	0.08	−0.51	0.613	ns
Actor effect significant other	−0.08	0.07	−1.06	0.294	Partner effect significant other	−0.27	0.07	−3.69	< 0.001^**^	0.37
Partner effect worker	−0.04	0.07	−0.54	0.589						
Partner effect significant other	−0.29	0.08	−3.77	< 0.001^**^						
Concern of the worker (*N* = 93)										
Intercept worker	3.06	0.13	23.61	< 0.001^**^	Intercept	3.01	0.12	25.19	< 0.001^**^	
Intercept significant other	2.98	0.13	23.22	< 0.001^**^	Actor effect	−0.14	0.04	−3.84	< 0.001^**^	0.30
Actor effect worker	−0.22	0.06	−3.75	< 0.001^**^	Partner effect	−0.15	0.04	−4.02	< 0.001^**^	0.32
Actor effect significant other	−0.04	0.07	−0.57	0.567						
Partner effect worker	−0.08	0.07	−1.18	0.242						
Partner effect significant other	−0.25	0.06	−4.38	< 0.001^**^						
Illness coherence (*N* = 93)										
Intercept worker	3.12	0.15	21.03	< 0.001^**^	Intercept	3.10	0.14	22.21	< 0.001^**^	
Intercept significant other	3.09	0.15	20.97	< 0.001^**^	Actor effect	−0.02	0.04	−0.43	0.672	ns
Actor effect worker	0.05	0.06	0.82	0.417	Partner effect	0.00	0.04	0.09	0.930	ns
Actor effect significant other	−0.09	0.07	−1.18	0.242						
Partner effect worker	−0.05	0.08	−0.67	0.505						
Partner effect significant other	0.07	0.06	1.16	0.250						
Emotional response of the worker (*N* = 93)										
Intercept worker	3.08	0.14	22.07	< 0.001^**^	Intercept	3.05	0.13	23.40	< 0.001^**^	
Intercept significant other	3.04	0.14	21.96	< 0.001^**^	Actor effect	−0.03	0.04	−0.71	0.481	ns
Actor effect worker	−0.05	0.07	−0.71	0.482	Partner effect	−0.12	0.04	−3.24	0.001^**^	0.23^c^
Actor effect significant other	0.01	0.07	0.22	0.826						
Partner effect worker	−0.09	0.07	−1.31	0.195						
Partner effect significant other	−0.16	0.07	−2.31	0.023^*^						

^a^unadjusted beta coefficients; ^b^beta coefficients adjusted for age; ^c^adjusted effect size in accordance with recommendations from Kenny, Kashy and Cook [22]; *p < 0.05; **p < 0.01; ns = non-significant; *N* = number of dyads included

#### Composite Illness Perceptions Score

**Fig. 2:**
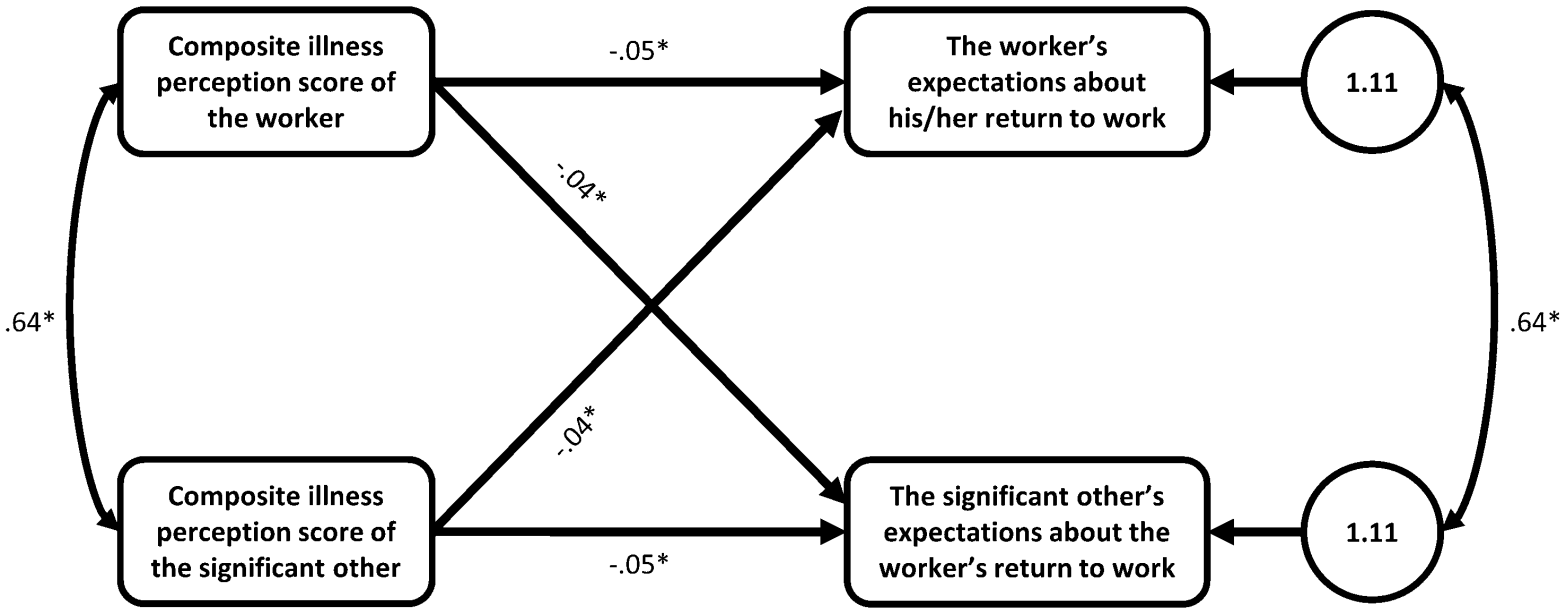
Final Actor-Partner Interdependence model with beta coefficients for the association between the illness perceptions score and expectations about the worker’s full RTW. *p < .05. As there were no statistically significant differences in effects between workers and significant others, the average actor and partner effects were estimated in the final model. Actor-Partner Interdependence Model depicting the average actor and partner effects of dyad members’ composite illness perception scores on their expectations about the worker's full return to work

Both actor and partner effects of illness perceptions on expectations about the worker’s RTW were identified in the two-intercept model. Contrast analysis showed that there were no statistically significant differences between workers and significant others with regard to the intercepts, actor effects and partner effects. The average actor effect (*B* = -0.05, *SD* = 0.01, *t*(168) = −5.80, *p* < 0.001) and average partner effect (*B* = −0.04, *SD* = 0.01, *t*(171) = −5.58, *p* < 0.001) were both significantly associated with RTW expectations of workers and significant others. In other words, the illness perceptions of workers and significant others were significantly associated with a dyad member’s own RTW expectations, as well as the expectations of the other dyad member. In this context, more negative illness perceptions were related to more negative expectations about the worker’s RTW. The effect sizes for the actor and partner effects were 0.37 and 0.35 respectively, reflecting medium sized effects. The final model is shown in Fig. 2.

#### Domains of Illness Perceptions

For most illness perception domains, we found small to moderate actor effects and partner effects on RTW expectations (*r*_*d*_ range: 0.23–0.44). For the domain *personal control of the worker*, only perceptions of significant others were significantly associated with expectations of workers (*B* = 0.19, *SD* = 0.05, *t*(87) = 3.61, *p* = 0.001) and significant others (*B* = 0.22, *SD* = 0.05, *t*(91) = 4.41, *p* < 0.001) about the worker’s RTW. For the domain *emotional response,* only a partner effect was found (*B* = −0.12, *SD* = 0.04, *t*(153) = −3.24, *p* = 0.001). There were no significant effects of dyad members’ perceptions about the worker’s *illness coherence* on expectations about RTW of the worker.

## Discussion

The results of this study show that most illness perceptions and RTW expectations are moderately to strongly correlated between workers with chronic diseases and their significant others, indicating that dyad members’ illness perceptions and RTW expectations are interdependent. Moreover, we found evidence that illness perceptions of workers and their significant others are associated with both their own and the other dyad member’s expectations (i.e., intrapersonal and interpersonal effects) about full RTW of the worker with the chronic disease. More specifically, within dyads of workers and significant others, more negative illness perceptions were related to more negative expectations on whether the sick-listed worker would be fully back at work in six months.

Our results are in line with prior studies reporting that illness perceptions of patients and their spouses are often similar and strongly correlated [18, 21, 37, 38]. For instance, Richardson et al. found positive correlations between cancer patients and caregivers for most illness perception domains [38]. Similar to our findings, other studies among patients and their spouses have found evidence of intrapersonal and interpersonal associations between illness perceptions and quality of life [38], perceptions of spouse undermining (i.e., negative reactions of the spouse towards the patient, such as criticism or anger) [37], and patients’ well-being [18]. Moreover, our results support previous qualitative studies that have suggested that not only the worker’s own perceptions and appraisals, but also the perceptions and appraisals of their significant others are important in the context of work participation and RTW [22, 23].

Our findings highlight the importance of interpersonal and dyadic processes in the development of illness perceptions and expectations about RTW and add to the empirical evidence regarding the role of significant others in this context. While this study does not provide insight into how and why illness perceptions and RTW expectations of workers and significant others are interrelated, as mentioned before, interactions between the worker and the significant other have been shown to play an important role in the development of illness perceptions and in how the worker and significant other adapt to the chronic disease [15–18]. Regarding this study, workers and significant others sharing information and discussing issues related to the worker’s illness and return to work could explain the strong interdependence between their illness perceptions and expectations about the worker’s RTW. Similarly, the interpersonal associations between illness perceptions and RTW expectations within dyads might be driven by responses and interactions elicited by the worker’s and significant other’s illness perceptions. For example, triggered by negative perceptions about the disease, a significant other might respond solicitously toward the worker (e.g., encourage resting, discouraging RTW), which could in turn negatively affect the worker’s RTW expectations. Similarly, a worker’s negative illness perceptions could lead to maladaptive or unhelpful illness behaviors such as catastrophizing or withdrawing from activities [39, 40], which can lead to negative RTW expectations of the significant other.

### Strengths and Limitations

The strength of this study is reflected in its dyadic design, which enabled us to extend previous literature on the intrapersonal associations between illness perceptions and RTW expectations to the interpersonal level. Applying the APIM framework allowed us to study both intrapersonal and interpersonal associations while taking the dyad members’ interdependence into account. A limitation of this study is that no causal effects between illness perceptions and RTW expectations could be tested, as we used an observational cross-sectional design. Another limitation is that some selection bias seems to have occurred, possibly limiting the generalizability of our study findings. More specifically, compared to a representative cohort of workers with a chronic disease from Arbo Unie, the mean age in our sample was considerably higher, and a relatively high percentage of workers in our study was male and had a musculoskeletal disorder or a mental illness. In addition, most participants in our study rated the quality of their relationship with the other dyad member with an eight or above, which might indicate that workers and significant others who were less satisfied with their relationship were less inclined to participate in this study. This selection bias may have influenced our results if dyadic processes differ depending on the type of disease, relationship satisfaction, age or gender. For instance, as relationship satisfaction has been shown to be positively associated with similarity of illness representations of patients with chronic diseases and their partners [41], it is possible that the illness perceptions and RTW expectations were more similar in our study than among workers and significant others who are less satisfied with their relationship.

### Practical Implications

The findings of this study add to our understanding of the dyads’ role in RTW by indicating that illness perceptions and RTW expectations are probably the result of a dyadic process between workers and their significant others. Our findings confirm the importance of addressing illness perceptions and RTW expectations of the sick-listed worker and suggest that occupational health professionals should also assess illness perceptions and RTW expectations of significant others. An assessment of RTW expectations of both workers and their significant others could help occupational health professionals to identify workers at risk of long-term sickness absence [42]. In addition, exploring whether illness perceptions of workers and their significant others play a role can provide insight into inadequate or maladaptive perceptions and coping strategies that may be modified to achieve more realistic RTW expectations and facilitate sustainable RTW. This might be especially useful in situations in which the RTW expectations are unrealistically positive or negative and markedly different from the expectations of the occupational health professional. In this context, occupational health professionals could use the revised or brief version of the IPQ to explore and discuss illness perceptions of workers and significant others [43, 44]. Furthermore, occupational health professionals could consult with the worker and the significant other to assess their illness perceptions and RTW expectations and modify inadequate or maladaptive perceptions by providing information about the worker’s disease and RTW process [43–47]. If appropriate, occupational health professionals could refer the worker and significant other to other health care providers such as a psychologist, social worker, or medical specialist to intervene on inaccurate and maladaptive illness perceptions [43–47].

### Recommendations for Future Research

While prior research has shown that a worker’s expectations about RTW is an important prognostic factor of RTW, more research is needed to investigate the intrapersonal and interpersonal associations of illness perceptions and RTW expectations of workers and their significant others with actual RTW. In addition, more research is needed to explore the pathways through which illness perceptions are related to RTW expectations and actual RTW. For instance, future research might investigate the relationship between illness perceptions within dyads and duration of sick leave, and whether this relationship is mediated by RTW expectations of workers and their significant others. Furthermore, additional research is needed to determine whether the interpersonal associations of illness perceptions with RTW expectations differ depending on the disease and the type of relationship between the worker and his or her significant other. For example, prior research suggests that living together with a partner and the way patients and their partners interact with each other in their shared daily life play an important role in the functioning of patients with chronic diseases [48]. It is therefore likely that the interpersonal associations between illness perceptions and RTW expectations are stronger for dyads in which the significant other is the worker’s partner rather than a family member or friend not living with the worker. In addition, more research is needed to obtain additional information on how and why illness perceptions and RTW expectations of workers and significant others are interrelated as this could provide valuable insight into how significant others could be involved in the RTW process of sick-listed workers. Such research might use a dyadic diary approach to gain insight into how verbal and non-verbal communication between workers and significant others relate to their illness perceptions and RTW expectations. Finally, future research should focus on the development and evaluation of interventions aimed at promoting adaptive illness perceptions and RTW expectations in dyads of workers with chronic diseases and their significant others.

## Conclusion

This study adds to our understanding of the dyads’ role in the RTW process by indicating that illness perceptions and RTW expectations are likely to be the result of a dyadic process between workers and their significant others. When trying to facilitate adaptive illness perceptions and RTW expectations to support sustainable RTW, involving significant others may be more effective than an individualistic approach targeted at the worker only.

## Supplementary Information

Below is the link to the electronic supplementary material.

Supplementary file1 (DOCX 21 KB)

## Data Availability

The dataset generated and/or analyzed during the current study is not publicly available, but is available from the corresponding author upon reasonable request.

## Abbreviations

RTW: Return to work
APIM: Actor-Partner Interdependence Model
OHS: Occupational health service
IPQ-B: Brief Illness Perception Questionnaire
