# Supplementary material for: The Associations Between Illness Perceptions and Expectations About Return to Work of Workers With Chronic Diseases and Their Significant Others: A Dyadic Analysis
Source: J Occup Rehabil. 2022 Aug 17;33(1):189–200. doi: 10.1007/s10926-022-10062-7 (PMC10025207; doi:10.1007/s10926-022-10062-7)
Supplement: Supplementary file 1 — Supplementary file1 (DOCX 21 KB) [file 10926_2022_10062_MOESM1_ESM.docx]

**Article title:** The associations between illness perceptions and expectations about return to work of workers with chronic diseases and their significant others: a dyadic analysis

**Journal:** Journal of Occupational Rehabilitation

**Authors:** drs. N.C. Snippen^1^, dr. H.J. de Vries^1^, prof. C.A.M. Roelen^1,2^, prof. S. Brouwer^1^, prof. M. Hagedoorn^3^

**Corresponding author:** N.C. Snippen, University of Groningen, University Medical Center Groningen, Department of Health Sciences, Community and Occupational Medicine, Groningen, The Netherlands, [n.c.snippen@umcg.nl](mailto:n.c.snippen@umcg.nl)

**Online Resource 1.** Syntax for the first three steps of the APIM analyses to determine the dyadic associations between illness perceptions and RTW expectations

| **Analyses step** |  | **Syntax** |
| --- | --- | --- |
| Step 1: Estimating the full APIM |  | **MIXED**  RTW_expectations_A **BY** partnum **WITH** IPQ_A IPQ_P age_A  **/FIXED=**partnum partnum* IPQ_A partnum* IPQ_P partnum* age_A **\| NOINT**  /**PRINT=SOLUTION TESTCOV**  **/REPEATED**=partnum \| **SUBJECT**(DyadID) **COVTYPE**(CSH). |
| Step 2: Testing for differences between dyad members |  | **/TEST** 'Main Effect for partnum' partnum -1 1  **/TEST** 'Interaction Effect for Actor*partnum' partnum* IPQ_A -1 1  **/TEST** 'Interaction Effect for Partner*partnum' partnum* IPQ_P -1 1 |
| Step 3: Estimating average intercepts and effects across dyad members |  | **/TEST** 'Average intercept' partnum .5 .5  **/TEST** 'Average Actor Effect' partnum* IPQ_A .5 .5  **/TEST** 'Average Partner Effect' partnum* IPQ_P .5 .5 |

RTW_expectations = return to work expectations; IPQ = illness perceptions score; A = actor; P = partner; partnum = identifier of the dyad member (i.e., worker vs. significant other)
